# The Role of Macrophage Efferocytosis in the Pathogenesis of Apical Periodontitis

**DOI:** 10.3390/ijms25073854

**Published:** 2024-03-29

**Authors:** Xiaoyue Guan, Yuting Wang, Wenlan Li, Wenli Mu, Yifei Tang, Mingfei Wang, Abdelrahman Seyam, Yao Yang, Lifei Pan, Tiezhou Hou

**Affiliations:** 1 Key Laboratory of Shaanxi Province for Craniofacial Precision Medicine Research, College of Stomatology, Xi’an Jiaotong University, Xi’an 710004, Chinaa.seyam96@gmail.com (A.S.);; 2 Clinical Research Center of Shaanxi Province for Dental and Maxillofacial Diseases, College of Stomatology, Xi’an Jiaotong University, Xi’an 710004, China; 3 Department of Cariology and Endodontics, College of Stomatology, Xi’an Jiaotong University, Xi’an 710004, China

**Keywords:** apical periodontitis, macrophage efferocytosis, animal model, ARA290

## Abstract

Macrophages (Mφs) play a crucial role in the homeostasis of the periapical immune micro-environment caused by bacterial infection. Mφ efferocytosis has been demonstrated to promote the resolution of multiple infected diseases via accelerating Mφ polarization into M2 type. However, the Mφ efferocytosis–apical periodontitis (AP) relationship has not been elucidated yet. This study aimed to explore the role of Mφ efferocytosis in the pathogenesis of AP. Clinical specimens were collected to determine the involvement of Mφ efferocytosis in the periapical region via immunohistochemical and immunofluorescence staining. For a further understanding of the moderator effect of Mφ efferocytosis in the pathogenesis of AP, both an in vitro AP model and in vivo AP model were treated with ARA290, a Mφ efferocytosis agonist. Histological staining, micro-ct, flow cytometry, RT-PCR and Western blot analysis were performed to detect the inflammatory status, alveolar bone loss and related markers in AP models. The data showed that Mφ efferocytosis is observed in the periapical tissues and enhancing the Mφ efferocytosis ability could effectively promote AP resolution via facilitating M2 Mφ polarization. Collectively, our study demonstrates the functional importance of Mφ efferocytosis in AP pathology and highlights that accelerating Mφ efferocytosis via ARA290 could serve as an adjuvant therapeutic strategy for AP.

## 1. Introduction

Apical periodontitis is a common oral inflammatory disease primarily caused by invasive anaerobic bacteria from the dental pulp and root canals [1]. The constant infections eventually lead to the local inflammation and alveolar bone resorption in the periapical region [2]. Despite root canal treatment (RCT) being performed to remove the infectious substances from the root canal system, nearly 4–15% of teeth still experience AP pain or are even subject to extraction [3]. Cumulative evidence has put forward the notion that the interplay between pathogens and the host immune system in periapical regions accounts for RCT therapy resistance [4]. Thus, exploring the causal mechanisms involved in the pathogenesis of AP may be essential for the management of AP.

Recently, Mφ efferocytosis has been reported to influence Mφ polarization [5,6,7]. Mφ efferocytosis is defined as a process where the apoptotic neutrophils in diseased tissues are engulfed by Mφs (defined as efferocytes) [8]. Notably, after the clearance of apoptotic neutrophils, the efferocytes release various biologically active signaling molecules and extracellular vesicles, which are then taken up by neighboring or distant Mφs to effect phenotypic change, including accelerating Mφs polarized to the M2 phenotype [5]. The activation of Mφ efferocytosis is composed of three stages [9,10]. Firstly, the apoptotic neutrophils in diseased regions spontaneously release the “find me” signals to recruit Mφs. Then, the aggregated Mφs recognize the apoptotic neutrophils via their characteristic cell surface changes. The most familiar cell surface change is the exposure of phosphatidylserine (PS), a critical “eat me” signal, on the surface of dying cells [11]. In addition, MerTk (C-mer proto-oncogene tyrosine kinase), the key receptor on the surface of Mφs, can recognize and bind to PS [11]. Moreover, Gas6, a bridging protein, is responsible for bridging PS indirectly with efferocytosis-related receptors on Mφs’ surface [12]. Once finishing the binding step, the apoptotic neutrophils start to be swallowed and degraded by the efferocytes, which can accelerate the polarization of Mφs toward a pro-resolving M2 phenotype [5].

In view of the crucial role of Mφ efferocytosis on apoptotic cells’ clearance and Mφ polarization, it may not be surprising that Mφ efferocytosis is involved in the etiology and pathogenesis of multiple inflammatory diseases such as atherosclerosis (AS), osteoarthritis (OA) and periodontitis [13,14,15,16]. Lv et al. demonstrated that increasing the Mφ efferocytosis ability in an AS model in ApoE^−/−^ mice could effectively suppress AS progression, even leading to the resolution of AS [17]. Moreover, Sordo et al. found that there was a marked reduction in the fraction of synovial tissue Mφs engaging in efferocytosis in patients with late-stage knee OA, resulting in the accumulation of apoptotic synovial cells and then exacerbating the disease activity in OA patients [18]. In order to further understand the role of efferocytosis in OA, Yao et al. increased the Mφ efferocytosis ability in an OA mice model via the intra-articular injection of Gas6, and then confirmed that targeting Mφ-associated efferocytosis could significantly suppress OA progression [14]. Particularly, research given by Kourtzelis et al. and Li et al. delineated the relation between efferocytosis and periodontitis [15,16]. They unveiled that Mφ efferocytosis participates in the progression of periodontitis. The regulation of Mφ efferocytosis is closely correlated with inflammation resolution in periodontal tissues [15,16]. Taken above, Mφ efferocytosis highlights great capacities to suppress the deterioration of inflammatory diseases or accelerate the resolution of inflammation. However, the role of Mφ efferocytosis in the pathogenesis of AP is still elusive. Therefore, this study aims to outline the evidence for the putative involvement of macrophage efferocytosis in the pathogenesis of AP.

In the current study, we used human periapical tissues as well as a cell model to investigate the involvement of Mφ efferocytosis in AP. Next, we constructed a co-cultured cell model and a mouse model of AP, with the application of efferocytosis agonist (ARA290), to explore the role of Mφ efferocytosis in attenuating AP progression. Deeper investigations and understanding into the role of efferocytosis in AP can provide therapeutic targets for inflammation resolution and tissue regeneration.

## 2. Results

### 2.1. Observation of Inflammatory Status in Periapical Tissues

**Figure 1:**
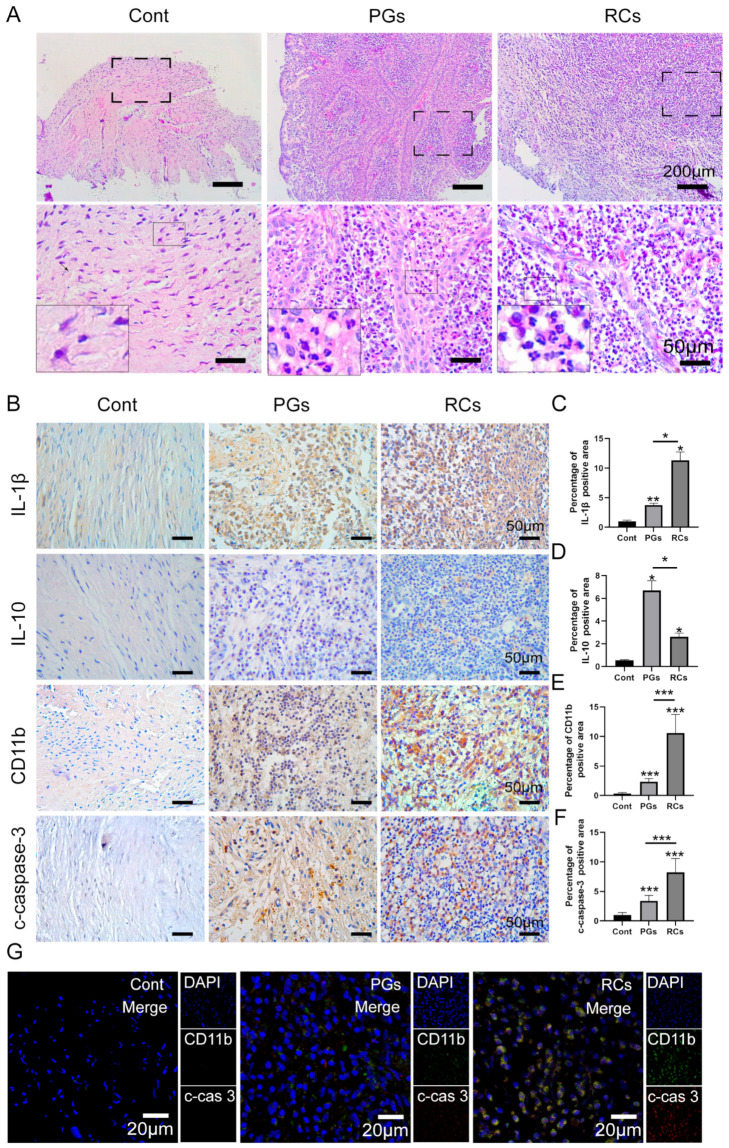
The infiltration of apoptotic neutrophils in the periapical regions. (**A**) Hematoxylin and eosin staining (scale bar = 200 μm) showing inflammatory infiltrates under different magnifications in healthy control tissues, PGs and RCs. (**B**) Immunohistochemical staining (scale bar = 50 μm) of Interleukin (IL)-1β, IL-10, CD11b and c-caspase-3 in the periapical region of healthy individuals and those with PGs and RCs. (**C**–**F**) The quantification of (**C**) IL-1β, (**D**) IL-10, (**E**) CD11b and (**F**) c-caspase-3 in each group. The results are expressed as the area of double-positive cells/mm^2^; * *p* < 0.05, ** *p* < 0.01, *** *p* < 0.001 (**G**) CD11b-c-caspase-3 double labeling (scale bar = 20 μm) in each group. Cont, healthy control; PGs, periapical granulomas; RCs, radicular cysts; c-cas3, c-caspase3.

HE (hematoxylin–eosin) staining revealed that periapical tissues with AP exhibited a greater infiltration of inflammatory cells compared to healthy periapical tissues (Figure 1A). Immunohistochemical staining demonstrated a higher expression of IL-1β and IL-10 in apical periodontitis (Figure 1B–D). Specifically, IL-1β expression was higher in radical cysts (RCs) than in periapical granulomas (PGs) (Figure 1B,C), while the expression of IL-10 was stronger in PGs than in RCs (Figure 1B,D). Furthermore, we conducted an additional investigation into neutrophil infiltration and apoptosis in the periapical area. Little neutrophil infiltration (CD11b^+^) could be observed in healthy periapical tissues. However, in the periapical lesions, particularly in the RCs group, there was a notable increase in the number of CD11b^+^ cells (Figure 1B,E). Next, we found that the expression of cleaved-caspase-3 (c-caspase-3), a marker of cell apoptosis, was significantly increased in inflamed periapical lesions when compared to the control group (Figure 1B,F). Notably, the quantification of c-caspase-3 positive cells in the RCs group was higher than those in the PGs group (Figure 1B,F). Furthermore, immunofluorescence co-staining exhibited a higher c-caspase-3 expression in the infiltrated neutrophils (CD11b^+^) of apical periodontitis (Figure 1G). The results revealed that with the increased co-localization of CD11b^+^ and c-caspase-3 in the periapical region, the expression of IL-1β was elevated correspondingly, but the expression of IL-10 was decreased gradually (Figure 1B). Thus, the above data indicated that the accumulation of apoptotic neutrophils in periapical lesions intensified the inflammatory status in the periapical region. The timely cleaning of apoptotic neutrophils in periapical lesions may restrain AP progression.

### 2.2. Macrophage Efferocytosis in Periapical Tissues

**Figure 2:**
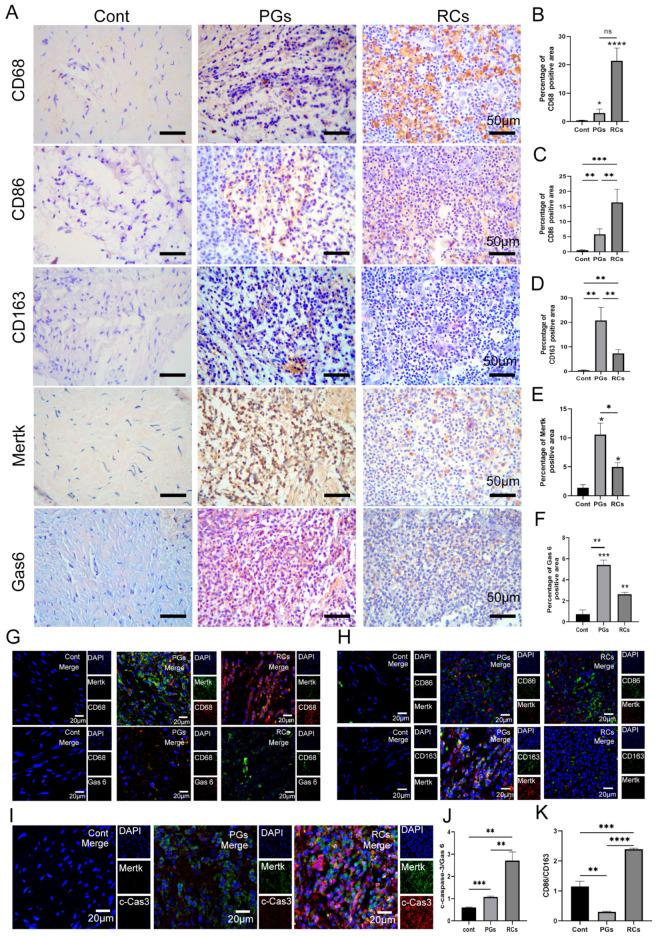
Macrophage efferocytosis and polarization in the periapical regions. (**A**) Immunohistochemical staining (scale bar = 50 μm) of Mertk, Gas6, CD68, CD86 and CD 163 in the periapical region of healthy individuals and those with PGs and RCs. (**B**–**F**,**K**) The quantification of (**B**) CD68, (**C**) CD86, (**D**) CD163, (**E**) Mertk, (**F**) Gas6 and (**K**) M1/M2 ration in each group. The results are expressed as the area of immune-positive cells/mm^2^. (**G**) Mertk-CD68 and Gas6-CD68 double labeling (scale bar = 20 μm) in each group. (**H**) Mertk-CD86 and Mertk-CD163 double labeling (scale bar = 20 μm) in each group. (**I**,**J**) Mertk-Cas3 double labeling (scale bar = 20 μm) in healthy control group, PGs and RCs region. * *p* < 0.05, ** *p* < 0.01, *** *p* < 0.005, **** *p* < 0.001, ns. *p* > 0.05. Cont, healthy control; PGs, periapical granulomas; RCs, radicular cysts; c-cas3, c-caspase3.

In addition to neutrophils, abundant Mφs were observed in the periapical lesions simultaneously. Immunohistochemistry (IHC) staining uncovered that little Mφ infiltration (CD68^+^) could be found in the Cont group, whereas the number of CD68^+^ cells were substantially higher in the periapical lesions, especially in the RCs group (Figure 2A,B). Notably, the infiltration of the M1 sub-type Mφs (CD86^+^), M2 sub-type Mφs (CD163^+^) and M1/M2 ratio in apical periodontitis was significantly higher than that in the Cont group (Figure 2A,C,D,K). Furthermore, in comparison with the PGs group, more CD86^+^ cells and M1/M2 ration were observed in the RCs group, but fewer CD163^+^ cells were found in the RCs group (Figure 2A,C,D,K). Next, we detected the expression of Mertk and Gas6, the marker of efferocytosis, within the periapical tissues. IHC staining revealed that the expression of Mertk and Gas6 in inflamed tissues was higher than that in the healthy control, and the expression of Mertk and Gas6 reduced markedly in the RCs when compared with the PGs (Figure 2A,E,F). In addition, IF staining further detected the expression of Mertk and Gas6 within macrophages in the periapical regions and disclosed a strong co-localization of Mertk and CD68^+^ cells in inflamed periapical sites than healthy tissues, whereas a weak co-localization of Gas6 and CD68^+^ cells was observed (Figure 2G). Interestingly, the expression pattern of Mertk in Mφ is in accordance with CD163^+^ cells, that is, the expression of Mertk in the PGs group was higher than that in the RCs group (Figure 2A,H). Moreover, immunofluorescence (IF) co-staining was performed to investigate the expression of both Mertk and c-caspase-3 in the periapical region. The results revealed that the ratio of c-caspase-3 to Mertk was higher in periapical lesions, especially in the RCs groups (Figure 2I,J). In summary, these results hint that the reduced phagocytosis of apoptotic neutrophils by Mφs may account for the exacerbation of apical periodontitis.

### 2.3. The Changes in Macrophage Efferocytosis Efficiency Were Observed In Vitro under Inflammatory Condition

**Figure 3:**
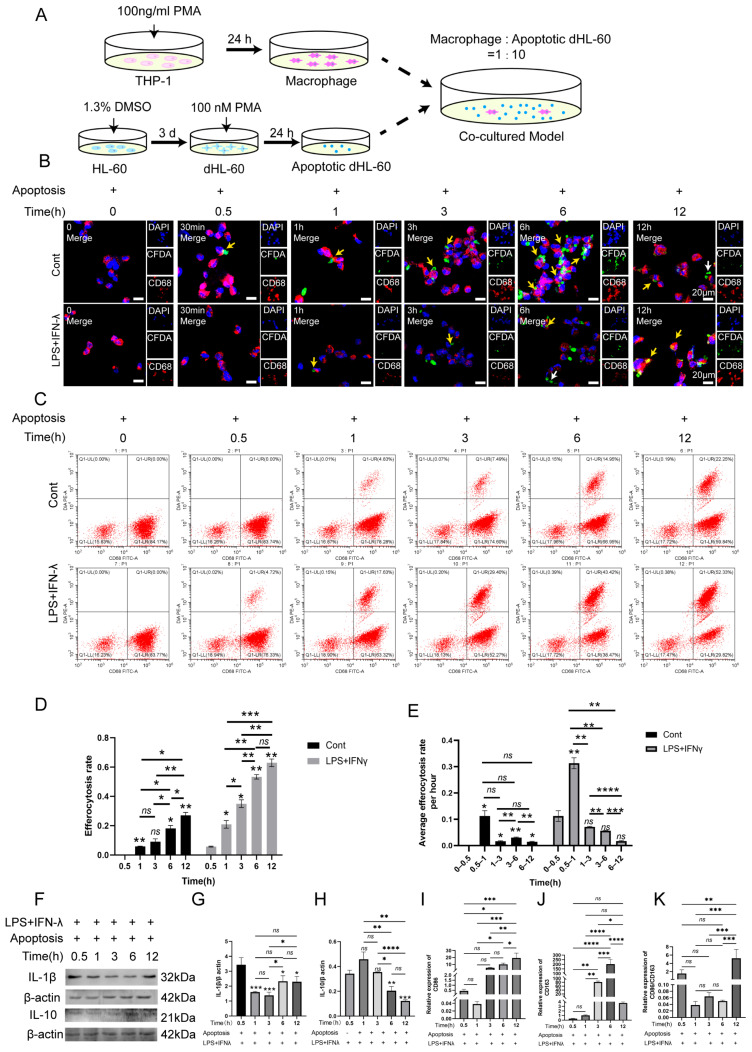
The correlation with macrophage efferocytes and macrophage polarization in inflammatory condition. (**A**) The schematic drawing of the co-culturing cell model. (**B**) The co-cultured cell model of apoptotic neutrophils and macrophages under normal and Pg. LPS+IFN-γ-stimulated condition. IF staining was conducted to detect the efferocytosis capacity. Yellow arrow: the engulfment of apoptotic cell by macrophages; white arrow: the apoptotic cell that was not engulfed by macrophages. Green, apoptotic neutrophils; red, macrophages; blue, DAPI. Scale bar = 20 μm. (**C**) The co-cultured cell model of apoptotic neutrophils and macrophages under normal and Pg. LPS+IFN-γ-stimulated condition detected by flow cytometry. The upper right quadrant: PKH26^+^ and CD68^+^ cells, which represent the macrophages that engulf the apoptotic neutrophils. The lower right quadrant: PKH26^−^ and CD68^+^ cells, which refer to the macrophages that did not engulf the apoptotic neutrophils. (**D**,**E**) The quantification of efferocytosis rate and average efferocytosis rate per hour. (**F**–**H**) Representative immunoblotting images and quantification of IL-10 (**F**,**H**), IL-1β (**F**,**G**) in co-cultured cell model under normal and inflamed conditions. (**I**–**K**) The expression of CD86 and CD163 was detected via RT-PCR. (**K**) The M1/M2 ration was calculated according to the expression of CD86 and CD163. * *p* < 0.05, ** *p* < 0.01, *** *p* < 0.005, **** *p* < 0.001, ns. *p* > 0.05.

During this experiment, HL-60 cells were differentiated into neutrophils via stimulation with DMSO for 3 days. Then, 100 nM PMA was added into neutrophils for 24 h to establish apoptotic cell models (Figure S1). In comparison with the none-treated group, 100 nM PMA could cause neutrophil apoptosis (Figure S1). Subsequently, the Mφs were co-cultured with apoptotic neutrophils with or without inflammatory stimulation for different time points as follows: 0 h, 0.5 h, 1 h, 3 h, 6 h and 12 h. The co-culturing cell model is shown in Figure 3A. IF staining showed the Mφs’ recognition of apoptotic neutrophils occurred 0.5 h, 1 h, 3 h, 6 h and 12 h after the cells’ co-cultivation in both the normal condition (Cont) and inflammatory condition (AP) (Figure 3B). The efficiency of Mφ efferocytosis increased gradually in the Cont group and AP group from 0.5 h to 6 h, and then experienced a downward trend from 6 h to 12 h (Figure 3B). Moreover, from 0.5 h to 12 h, the efficiency of Mφ efferocytosis in the AP group was greatly weakened when compared with the Cont group. Flow cytometry further confirmed the above findings (Figure 3C–E). Next, Western blot analysis and RT-PCR were performed to detect the expression of IL-1β, IL-10, CD86 and CD163 in the AP group (Figure 3E–J). The results found that with the increased efficiency of Mφ efferocytosis, from 0.5 h to 6 h, the expression of IL-1β and CD86 decreased by degrees, whereas the activation levels of IL-10 and CD163 were elevated in parallel (Figure 3E–I). The ratio of CD86 to CD163 was analyzed and displayed a negative correlation with the macrophage efferocytosis efficiency (Figure 3J). Similarly, from 6 h to 12 h, as the capacity of Mφ efferocytosis weakened, the protein levels of CD86 and IL-1β, and the M1/M2 ration gradually improved; however, the expression of CD163 and IL-10 decreased correspondingly (Figure 3F–K). These results indicated that the inflammatory condition impaired the Mφ efferocytosis ability, and Mφ efferocytosis may decelerate the inflammatory status by promoting the generation of M2 Mφ and secreting anti-inflammatory cytokines.

### 2.4. Facilitating Macrophage Efferocytosis in Promoting M2 Sub-Type Macrophage Polarization

**Figure 4:**
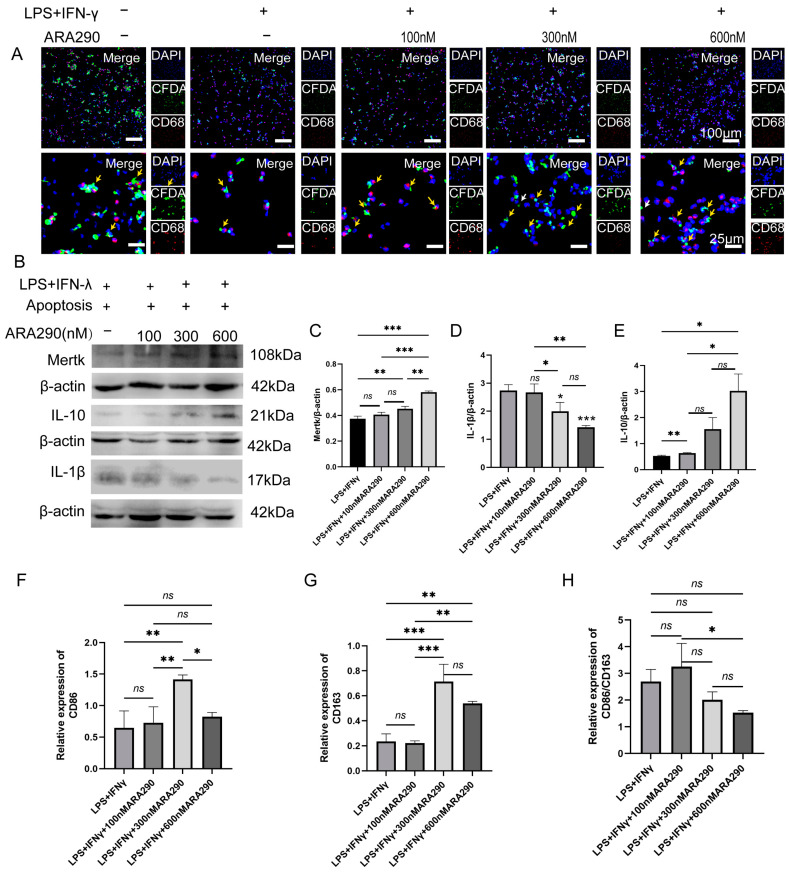
The enhanced capacity of macrophage efferocytosis could promote M2 macrophage polarization in inflammatory conditions. (**A**) The co-cultured cell model of apoptotic neutrophils and macrophages under Pg. LPS+IFN-γ-stimulated conditions with treatment of 100 nM, 300 nM and 600 nM ARA290 detected by immunofluorescence staining. Yellow arrow: the engulfment of apoptotic cell by macrophages; white arrow: the apoptotic cells that had not been engulfed by macrophages. Green, apoptotic neutrophils; red, macrophages; blue, DAPI. Scale bar of upper figure = 100 μm, scale bar of lower figure = 25 μm. (**B**–**E**) Representative immunoblotting images of different antibodies as indicated and their quantification. (**F**,**G**) The expression of CD86 and CD163 was detected via RT-PCR. (**H**) The M1/M2 ration was calculated according to the expression of CD86 and CD163. * *p* < 0.05, ** *p* < 0.01, *** *p* < 0.001, ns. *p* > 0.05.

To further confirm the effect of Mφ efferocytosis in resolving periapical inflammation, the Mφs were pre-treated with different doses of ARA290 under inflammatory conditions. ARA 290 has been defined as a small-molecule agonist of Mφ efferocytosis. As the concentration of ARA290 increased, the engulfment ability of Mφs to apoptotic neutrophils enhanced simultaneously (Figure 4A). Moreover, the expression of Mertk and IL-10 increased in a dose-dependent manner with the ARA290 concentration (Figure 4B,E). However, ARA290 decreased the expression of IL-1β (Figure 4B,D). Moreover, the expression of CD86 and CD163 increased to the peak with the ARA290 (300 μm) treatment, and then gradually reduced (Figure 4F,G). Although the ARA290 treatment increased the expression of both CD86 and CD163 simultaneously, the CD86/CD163 ration decreased significantly under the treatment with ARA290 at a 300 μm and 600 μm concentration (Figure 4H). All of the data suggested that ARA290 could promote the generation of M2-polarized Mφs and the secretion of IL-10 via enhancing the ability of Mφs efferocytosis under inflammation conditions.

### 2.5. ARA290 Reduced Alveolar Bone Loss in Periapical Lesions

**Figure 5:**
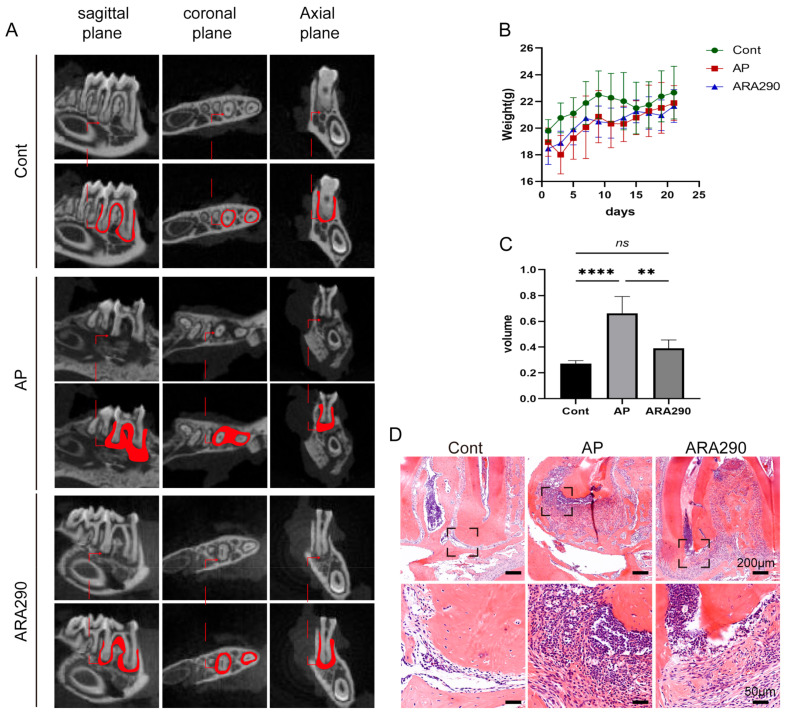
ARA290, the agonist of macrophage efferocytosis, could suppress apical periodontitis progression. (**A**) Representative radiographs of mandible first molar of experimental apical periodontitis. The images were taken using three-dimensional micro-Ct at three different views: coronal, sagittal and horizontal images. (**B**) The body weight of mice in different groups. (**C**) Quantification of bone loss volume in each group. ** *p* < 0.01, **** *p* < 0.001, ns. *p* > 0.05 (**D**) HE staining displayed bone loss and inflammatory status in periapical tissue in different group set. Scale bar of upper figure = 200 μm, scale bar of lower figure = 50 μm.

Firstly, micro-ct scanning and HE staining showed that in comparison with the control group, the AP group exhibited a radiolucency area around the root apex foramen, which demonstrated the successful establishment of the AP model in mice (Figure 5A,C,D). Furthermore, the effect of ARA290 in inhibiting alveolar bone loss was evaluated with micro-ct and HE staining experiments. Firstly, the body weight of mice in each group was recorded, and the results showed that there was no significant difference among these three groups (Figure 5B). Next, as shown in Figure 5A,C,D, ARA290 could effectively suppress the alveolar bone loss in periapical lesions. To our expectations, the results of the HE staining are consistent with the micro-ct outcome. Collectively, these findings demonstrated that ARA290 could ameliorate the alveolar bone loss that happened in the progression of AP.

### 2.6. ARA290 Alleviated the Progression of AP via Promoting Macrophage Efferocytosis

**Figure 6:**
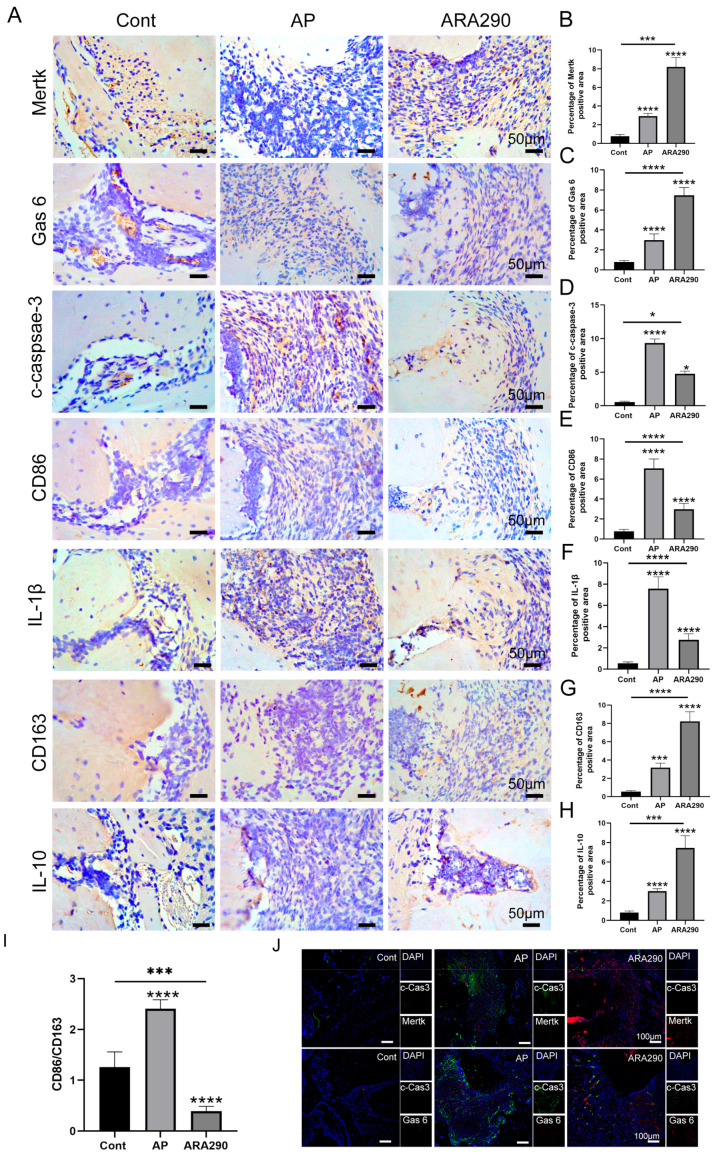
ARA290 promotes M2 macrophage polarization in periapical region. (**A**–**H**) The expression and quantification of Mertk, Gas6, c-caspase-3, CD68 and IL-1β in periapical tissue in different groups. Scale bar = 50 μm. (**I**) The M1/M2 ration was calculated according to the expression of CD86 and CD163. * *p* < 0.05, *** *p* < 0.005, **** *p* < 0.001 (**J**) Mertk-CD68 and Gas6-CD68 double labeling (scale bar = 20 μm) in each group. Green,c-Cas 3; red, Mertk/Gas 6; blue, DAPI.

The expression of Mφ efferocytosis-related protein, neutrophil apoptosis-related protein and the Mφ efferocytosis rate in mice periapical lesions was subsequently detected to identify the occurrence of Mφ efferocytosis in apical periodontitis. In comparison with the Cont group, the Mertk and Gas6 protein levels were slightly increased in the periapical lesions; however, the expression of c-caspase-3 was significantly raised in the inflamed sites (Figure 6A–D). Notably, the intraperitoneal injection of ARA290 in AP mice markedly increased the expression of Mertk and Gas6 in the periapical sites (Figure 6A–C). Furthermore, in comparison with the AP group, the AP+ARA290 group exhibited a higher expression of CD163 and IL-10, and a lower expression of CD86 and IL-1β in the periapical lesions (Figure 6E–H). Especially, the M1/M2 ration in the periapical lesions markedly reduced with the treatment of ARA290 (Figure 6I). In addition, the co-localization experiment observed a strong co-localization between Mertk and c-caspase-3, whereas there was a weak binding between Gas6 and c-caspase-3 (Figure 6J). Collectively, the above data demonstrated that Mφ efferocytosis was involved in the apical periodontitis progression in mice and promoting the efficiency of Mφ efferocytosis could facilitate the polarization of Mφ to the M2 profile, leading to the secretion of anti-inflammatory cytokines.

## 3. Discussion

Emerging evidence has been displayed regarding the effect of Mφ efferocytosis in suppressing inflammation progression and accelerating inflammation resolution [5,6,7]. In the current study, we discovered that Mφ efferocytosis participates in the AP pathology and promotes the resolution of AP by accelerating M2 Mφ polarization. Moreover, strengthening the capacity of Mφ efferocytosis could significantly ameliorate the inflammation status and bone loss in AP in in vitro and in vivo models. Notably, this study has initially observed the emergence of Mφ efferocytosis in human periapical tissues, further suggesting Mφ efferocytosis may be involved in AP pathogenesis.

Mφ efferocytosis represents the process of the timely and effective removal of undesirable cells, such as apoptotic neutrophils, by efferocytes [5]. This specialized process is of great importance for essential body functions, including immunoregulation, organism growth and the maintenance of tissue homeostasis [7]. During the valid process of Mφ efferocytosis, apoptotic neutrophils are cleared as they should be to block their harmful effects via avoiding their leakage to normal tissue microenvironments; or else, apoptotic neutrophils can be apt to secondary necrosis, leading to the release of detrimental autoantigens into healthy tissues [19]. Crucially, studies have elucidated that the impairment in the efferocytic course postponed inflammation resolution and aggravated the pathology in various inflammatory diseases, including neurodegenerative disorders, atherosclerosis, osteoarthritis and the like [18,20,21]. It is well known that Mφ efferocytosis facilitates the polarization of Mφs to an M2 phenotype, which stimulates the Mφ efferocytotic capacity in a feedback loop [22]. Cai et al. clarified that enhancing the efferocytosis efficiency of Pg.LPS-stimulated J774a.1 Mφs could markedly down-regulate the expression of proinflammatory cytokine TNF-α, whereas the expression of anti-inflammatory cytokine IL-10 was significantly up-regulated. Notably, the M1/M2 Mφ ration in the above cell model has also been down-regulated simultaneously, which suggested the regulatory effect of Mφ efferocytosis on Mφ polarization [23]. Moreover, Bhattacharya et al. further confirmed the role of efferocytosis on Mφ phenotype remodeling [5]. They depicted that after the engulfment of apoptotic neutrophils, the efferocytes secrete soluble mediators or extracellular vesicles, which can affect the Mφ phenotype remodeling in an autocrine/paracrine manner, promoting the polarization of Mφs toward a pro-resolving M2 phenotype [5]. In this study, we found that, in AP patients and the co-cultured cell model, as the AP progresses, the efferocytotic ability decreased (Figure 1 and Figure 2). Whereas, in comparison with the healthy periapical tissues, the protein levels of Gas6 and Mertk increased significantly in the AP clinic samples, including PGs and RCs. This may be attributed to the number of apoptotic neutrophils far outpacing the number of available efferocytes, leading to the periapical tissue suffering from continuous damage (Figure 2). Notably, as the efferocytosis rate increased in the inflamed co-cultured cell model, the expression of the anti-inflammatory cytokine (IL-10) up-regulated in parallel; however, the expression of the proinflammatory cytokine (IL-1β) and the ration of M1/M2 decreased correspondingly (Figure 4). This is particularly intriguing and raises the possibility that enhancing the Mφ efferocytosis could suppress the inflammation status and accelerate inflammation resolution in periapical lesions.

Previous data elucidated that ARA290 can promote the efferocytosis of Mφs in vitro, and the application of ARA290 enhances apoptotic cell uptake [24]. ARA290 is an erythropoietin-derived helix-B peptide and has been reported to retain the anti-inflammatory and tissue-protective functions of erythropoietin. Huang et al. claimed that ARA290 could reduce the serum concentrations of inflammatory cytokines IL-6, MCP-1 and TNF-α in systemic lupus erythematosus (SLE) mice [24]. Further, ARA290 suppressed the inflammatory activation of Mφs and promoted the uptake of apoptotic cells by Mφs, thus ameliorating SLE clinical and pathological manifestations [24]. Moreover, a study conducted by Xu et al. further validated the anti-inflammatory effect of ARA290. Daily administration of ARA290 could ameliorate depression-like behavior during chronic stress induction in mouse models via reducing neutrophils in the bone marrow and meninges [25]. Here, we tested the efferocytosis ability of Pg. LPS+IFN-γ-stimulated dTHP-1 with different concentrations of ARA290. According to our results, the expression of efferocytosis markers accelerated after treatment with ARA290 in a dose-dependent manner under the Pg.LPS + IFN-γ challenge (Figure 5). Moreover, the M1/M2 ration significantly decreased with the treatment of ARA290 under the 300 nM and 600 nM concentrations (Figure 5). Subsequently, we detected the role of ARA290-enhanced Mφ efferocytosis in regulating AP pathogenesis and uncovered that intraperitoneally administration restrained the inflammatory status and bone loss in periapical lesions (Figure 6). The promoting effect of Mφ efferocytosis on M2 Mφ polarization has been stressed before [26]. In consistency with a previous study, we found that ARA290 could accelerate the polarization of Mφs to the M2 phenotype and trigger the production of anti-inflammatory cytokines via enhancing the Mφ efferocytotic capacity in periapical lesions. Notably, a weak co-localization of Gas6 and c-caspase-3 was found in the ARA290-treated AP animal model when compared with the AP group; thus, we speculated that how ARA290 exerts its biological functions mainly depends on the Mertk signals. Moreover, in the ARA290-treated co-cultured cell model, we only detected the expression of Mertk, as the efferocytosis of THP-1 macrophages could not be transducted via Gas6 [27]. The above results imply that targeting Mφ efferocytosis reduces the inflammatory status and suppresses bone loss in the periapical region.

Considering the facilitating effect of Mφ efferocytosis on inflammation resolution, growing evidence has emerged to explore the biomarker that can target Mφ efferocytosis. Meriwether et al. revealed that Mφ cyclooxygenase 2 (COX2) could accelerate intestinal epithelial repair by mediating Mφ efferocytosis and efferocytosis-dependent reprogramming [28]. Moreover, developmental endothelial locus-1 (DEL-1) has also been demonstrated to regulate efferocytosis-induced Mφ reprogramming to a pro-resolving phenotype, promoting the resolution of experimental periodontitis [16]. Furthermore, in stroke mice, STAT6 activation in microglia and Mφs improved efferocytosis and modulated microglia/Mφ phenotype, accelerated inflammation resolution and ameliorated stroke outcomes [6]. This study has some limitations. In this study, we only focused on the involvement of Mφ efferocytosis in AP pathogenesis. Whereas more studies are being conducted by our research group, which aim at exploring the effective biomarkers that are capable of enhancing the Mφ efferocytosis capacity in AP. Moreover, the identification of efferocytes in in vitro or in vivo models mainly depends on indirect testing, including flow cytometry, IHC staining, IF staining, etc. In the future study, direct testing, such as electron microscopy (EM), should be conducted to observe the efferocytes in periapical lesions.

## 4. Materials and Methods

### 4.1. Clinic Samples Collection

The clinic apical periodontitis samples were collected from 18 subjects who were diagnosed with AP according to clinical and radiographic examination during endodontic microsurgery. Clinical manifestation covers a painful response to palpation or percussion or biting, no response to pulp vitality tests, fistula and sinus tract. In addition, the X-ray presented an apical radiolucency. Notably, the subjects were without any symptoms of acute AP. Healthy periapical tissues were obtained from 16 subjects who underwent permanent tooth extraction for orthodontic purposes. All subjects who enrolled in this study were free of systemic diseases and did not accept antibiotic treatment during the last 6 months. The collected tissues were fixed in 4% paraformaldehyde for 24 h and then embedded in paraffin. After that, the samples were consecutively sectioned into 4 μm slides for later experiments.

### 4.2. Induction of Mice AP Model and ARA290 Administration

In total, thirty male C57BL/6 mice with a body weight of 20–25 g (aged 6–8 w) were randomly assigned into 3 groups (*n* = 10/group): Control (Cont) group, apical periodontitis (AP) group and AP+ARA290 group. The mice were anesthetized with ketamine hydrochloride 10% (150 mg/kg body weight) and xylazine 2% (7.5 mg/kg body weight). Then, the bilateral first molar of the mice mandibles was perforated using a high speed 1/4# round bur until the pulp chamber was exposed [29]. Following the pulp operation, ARA290 (120 μg/kg, MCE, Monmouth Junction, NJ, USA) was intraperitoneally injected into mice three times per week [24]. Four weeks after AP induction, the mandible was isolated and fixed in 4% paraformaldehyde. The left side of the mandible was separated for micro-CT scanning and histology. The right hemimandibles were extracted for further immune testing. In this study, thirty C57BL/6 mice were kept in a regulated enviroment at a temperature of 25 °C, with access to food and water at all times and a 12 h light and dark cycle. All of the mice were managed sustainably to avoid mental and physical disorders.

### 4.3. Cell Culture and Treatments

Human monocytic leukemia THP-1 cells were purchased from Procell Life Science & Technology Co., Ltd. (Wuhan, China). Human promyelocytic leukemia cells (HL-60) were gifted from the Department of Hematology, the First Affiliated Hospical of Xi’an Jiaotong University (Xi’an, China). The above two cell lines were incubated in PRMI-1640 medium (Gibico, Billings, MT, USA) supplemented with 10% FBS (BI, Kibbutz, Beit Haemek, Israel) and cultured at 37 °C with 5% CO_2_ and 95% relative humidity. THP-1 cells were primed with 100 ng/mL phorbol 12-mysistate 12-acetate (PMA, Sigma-Aldrich, St. Louis, MO, USA) for 24 h to differentiate into Mφ (dTHP-1) [30]. Amounts of 100 ng/mL P. gingivalis lipopolysaccharide (Pg.LPS, Sigma-Aldrich, St. Louis, MO, USA) and 40 ng/mL interferon-γ (IFN-γ, Santa Clara, CA, USA) were then added into dTHP-1 to mimic the inflammatory conditions in the infected periapical region [31,32].

HL-60 cells were treated with 1.3% DMSO (Sigma-Aldrich, USA) for different time points; in detail, 1 d, 2 d, 3 d, 4 d and 5 d to differentiate into a neutrophil-like granulocyte (dHL-60). Western blot analysis was conducted to detect whether HL-60 was successfully differentiated into dHL-60. Next, in order to induce cell apoptosis, dHL-60 cells were further treated with 100 nM PMA for 24 h [33]. Flow cytometry was performed to confirm the apoptosis of dHL-60.

To investigate the phagocytic efficiency of Mφ on apoptotic cells under AP conditions, the Pg. LPS+IFN-γ-stimulated dTHP-1 cells were co-cultured with apoptotic dHL-60 cells, at a ratio of 1:10 for 0.5 h, 1 h, 3 h, 6 h and 12 h. To further explore the role of Mφ efferocytosis in AP pathology, the co-cultured cell model was simultaneously treated with ARA290 (#269143, MCE, Monmouth Junction, NJ, USA), a well-known efferocytosis agonist, under the following different ARA290 concentrations: 100 nM, 300 nM and 600 nM for 24 h.

### 4.4. Flow Cytometry

The rates of dHL-60 cell apoptosis were detected via flow cytometry (FCM) analysis using an Annexin V-FITC/PI Apoptosis kit (Bestbio, Nanjin, China). dHL-60 cells were seeded in a 6-well plate with 100 nM PMA for 24 h, then re-suspended in 400 μL binding buffer and stained with 10 μL of PI (propidium iodide) and 5 μL of FITC Annexin V. The treatments were continued for 16 min at 4 °C in the dark conditions. After that, the samples were submitted to a flow cytometer (Agilent, BD FACSCalibur, Santa Clara, CA, USA). Annexin V-positive and PI-negative dHL-60 cells were determined as the early apoptotic cells; furthermore, Annexin V- and PI-positive cells were identified as the late apoptotic cells.

The results were analyzed by FlowJo 1.6.0 software (Ashland, OR, USA).

### 4.5. Macrophage Efferocytosis Testing

To precisely explore the role of Mφ efferocytosis in the progression of AP, dHL-60 cells were firstly marked with fluorescence CFDA SE (Beyotime Biotechnology, Shanghai, China). Fluorescent dHL-60 cells were induced into apoptosis and then, the apoptotic dHL-60 cells were overlaid on the dTHP-1 (10:1) for 0.5 h, 1 h, 3 h, 6 h and 12 h at 37 °C under inflammatory conditions. Following efferocytosis, cells were rigorously washed three times with 1× ice-cold PBS to remove the dHL-60 cells that were not being engulfed. After that, the cells were fixed with 0.5% paraformaldehyde and incubated with CD68 antibody (1:100 dilution, Biolegend, San Diego, CA, USA) for 2 h at 37 °C to label dTHP-1. The efferocytotic efficiency was observed and analyzed under a confocal microscope (Olympus, Tokyo, Japan) at 400× magnification. Mφ efferocytosis was determined by counting cells including phagocytic green fluorescent apoptotic bodies. Data were represented as percent (%) efferocytosis–total number of cells with ingested apoptotic dHL-60 cells divided by the total number of dTHP-1 counted times 100.

Furthermore, flow cytometry was conducted for detecting the Mφ efferocytosis during the AP course. In brief, the apoptotic dHL-60 cells were marked with PKH26 (MCE, Monmouth Junction, NJ, USA) and were subsequently added to dTHP-1 cells under inflammatory conditions for different times as follows: 30 min, 1 h, 3 h, 6 h and 12 h. After being fixed with 0.5% paraformaldehyde, the co-cultured cell model was incubated with CD68-FITC antibody (1:100 dilution, Biolegend, Hangzhou, China). Then, the efferocytosis activity was measured via flow cytometry (BD FACSCalibur, CA, USA). Histograms were plotted using FlowJo^TM^ version 10 software (BD Biosciences, San Jose, CA, USA).

### 4.6. Micro-Ct

Fixed left hemimandibles were scanned as previously described using a cone beam-type tomograph (QuantumGX, PerkinElmer, Hopkinton, MA, USA). Scanning parameters were set as follows: voltage, 90 kV; current, 88 μA; FOV, 25 mm; voxel size, 50 μm. The scanned data were reconstructed and analyzed with Mimics 17.0 software (Materialize, Leuven, Belgium). Briefly, the lesion size was acquired via the subtraction of an averaged normal periodontal space in baseline controls from a total periapical radiolucent area and expressed as square millimeters. After micro-ct, the specimens were submitted to hematoxylin–eosin staining.

### 4.7. Hematoxylin–Eosin Staining

The decalcified left mandibles were embedded in paraffin and sectioned longitudinally at 4 μm thickness in mesio-distal orientation according to a general histology protocol. The slides were then dewaxed in xylene and rehydrated in gradient alcohols. Hematoxylin and eosin (HE) staining was performed on consecutive tissue sections. The slices with apical foramen were stained with HE according to the manufacturer’s manual (Solarbio, Beijing, China). Images were photographed and observed under light microscopy under 4× magnification (Olympus, Tokyo, Japan).

### 4.8. Immunohistochemical (IHC) or Immunofluorescence (IF) Staining

IHC and IF staining were conducted as a previous study described [34]. In brief, IHC staining was carried out by the Streptavidin–Biontin Complex (SABC) method on the basis of the manufacturer’s protocol (Boster, Wuhan, China). The sections were treated with digestive solution (Boster, Wuhan, China) to remove the antigen at 37 °C for 25 min. Thereafter, 3% hydrogen peroxide was added to the samples for endogenous peroxidase retrieval. Samples were then blocked with 5% BSA and incubated with the corresponding primary antibodies: anti-IL-1β (1:100 dilution, Santa Cruz, CA, USA), IL-10 (1:100 dilution, Santa Cruz, CA, USA), CD11b (1:100 dilution, Proteintech, Wuhan, China), c-caspase-3 (1:150 dilution, Bioss, Beijing, China), Mertk (1:100 dilution, Bioss, Beijing, China), Gas6 (1:100 dilution, Bioss, Beijing, China), CD68 (1:150 dilution, Bioss, Beijing, China), CD86 (1:150 dilution, Bioss, Beijing, China), CD163 (1:150 dilution, Bioss, Beijing, China). After 18 h, the slides were treated with secondary antibodies (Zhongshanjinqiao, Beijing, China) for 1.5 h. The immune reaction was observed with DAB substrate kit (Boster, Wuhan, China).

For the IF staining, after being blocked with 5% BSA, the tissue samples and the cell samples were incubated with the primary antibodies against CD11b and c-caspase-3, Mertk and CD68, Gas6 and CD68, Mertk and CD86, Mertk and CD163, Mertk and c-caspase3, Gas6 and c-caspase3. Of note, the dilution of the above primary antibodies was 1:150. Then, the secondary antibodies (dilution with 1:150, Boster, Wuhan, China) were goat against rabbit CY3 or goat against mouse FITC. IHC images were obtained using the Nikon microscope image system (Nikon Ltd., Tokyo, Japan). The immunology-positive cells were inspected and captured using a confocal microscope (Olympus, Tokyo, Japan) at 40× magnification. Semi-quantitative analysis was conducted using Image J software (Version 8, NIH, Bethesda, MD, USA). All of the experimental steps were taken by two independents in a double-blind manner.

### 4.9. Western Blot Analysis

All experiments were conducted in at least triplicates. The dTHP-1 cells were seeded at 5 × 10^4^ /well in 6-well plates with the stimulation of Pg.LPS and IFN-γ. Then, the apoptotic dHL-60 cells were added to the above cells to establish the co-cultured cell model. At the time points 0.5 h, 1 h, 3 h, 6 h and 12 h, after the co-cultured cell model was established, 150 μL RIPA lysis buffer containing 1 mM phenylmethanesulfonyl fluoride was added to the cells to obtain the cell lysate. Then, the supernatant was harvested after centrifuging at 12,000× *g* for 10 min at 4 °C. Thereafter, the supernatant was mixed with lading buffer (Boster, China) and boiled at 100 °C for 5 min. Equal amounts of protein (25 μg) were loaded on SDS-PAGE and electrophoresed at 60 V for 40 min, and then the voltage was switched to 110 V for 1 h. Then, the proteins were transferred onto polyvinylidene fluoride (PVDF) membranes (Millipore, St. Louis, MI, USA). The blots were then covered with primary antibodies against Mertk (1:300 dilution), IL-1β (1:300 dilution), IL-10 (1:300 dilution), β-actin (1:3000 dilution) at 4 °C for 18 h, followed by incubation with peroxidase-conjugated secondary antibodies. The immune blots were observed with enhanced chemiluminescence detection reagent (Millipore, St. Louis, MI, USA). Β-actin was set as the internal controls for the total protein. Image J software (Version 8, NIH, Bethesda, MD, USA)was operated to analyze the densitometry of the bands from the Western blot experiment. Notably, all of the primary antibodies were purchased from Bioss, China.

### 4.10. Reverse Transcription Quantitative Polymerase Chain Reaction (RT-qPCR)

Total RNA was extracted from the co-cultured cell model according to the TRIzol reagent protocol (Thermo Fisher Scientific, Waltham, MA, USA). The purity and the concentration of RNA was detected in a NanoDrop. Complementary dna (cDAN) was synthesized using RevertAid First Strand cDNA synthesis kit (Thermo Fisher Scientific, MA, USA), according to the manufacturer’s instructions. SYBR-green-based RT-PCR was performed with 50 cycles of 94 °C (1 min), 56 °C (1 min) and 72 °C (2 min) on an ABI PRISM 7700 (Applied Biosystems, Woburn, MA, USA). The analysis was conducted using the 2^−△△CT^ method. The primer sets used in this study are shown in Table 1.

### 4.11. Statistical Analysis

Statistical analysis was conducted using GraphPad Prism 8.0 software (GraphPad Software, Inc., La Jolla, CA, USA). All numerical data are presented as mean standard deviation. Multiple group comparisons were performed using a one-way analysis of variance (one-way ANOVA). *p* < 0.05 was considered statistically significant.

## 5. Conclusions

In summary, our in vivo and in vitro results suggested that Mφ efferocytosis is involved in the pathogenesis of AP. The above results provide a new perspective regarding the etiology and pathology of AP, indicating that promoting the efficiency of Mφ efferocytosis might be a new strategy for AP adjuvant treatment.

## Figures and Tables

**Table 1:** The primers for qRT-PCR.

Gene	Forward Primer (from 5′ to 3′)	Reverse Primer (from 3′ to 5′)
Gapdh	AGAAGGCTGGGGCTCATTTG	CTTCTGACACCTACCGGGGA
CD86	CTTCCTGCTCTCTGCTAACTTCA	TGCCAATGGGTCTTGGATTCT
CD163	CGGGAGATGAATTCTTGCCTGA	TGGGTCACTCGGAAATTCTATGG

## Data Availability

The datasets used or analyzed during the current study are available from the corresponding author upon reasonable request.

## Supplementary Materials

The following supporting information can be downloaded at: https://www.mdpi.com/article/10.3390/ijms25073854/s1.
