# Supplementary material for: The Role of Macrophage Efferocytosis in the Pathogenesis of Apical Periodontitis
Source: Int J Mol Sci. 2024 Mar 29;25(7):3854. doi: 10.3390/ijms25073854 (PMC11011522; doi:10.3390/ijms25073854)
Supplement: Supplementary file 1 [file ijms-25-03854-s001.zip › ijms-2909689-supplementary.pdf]

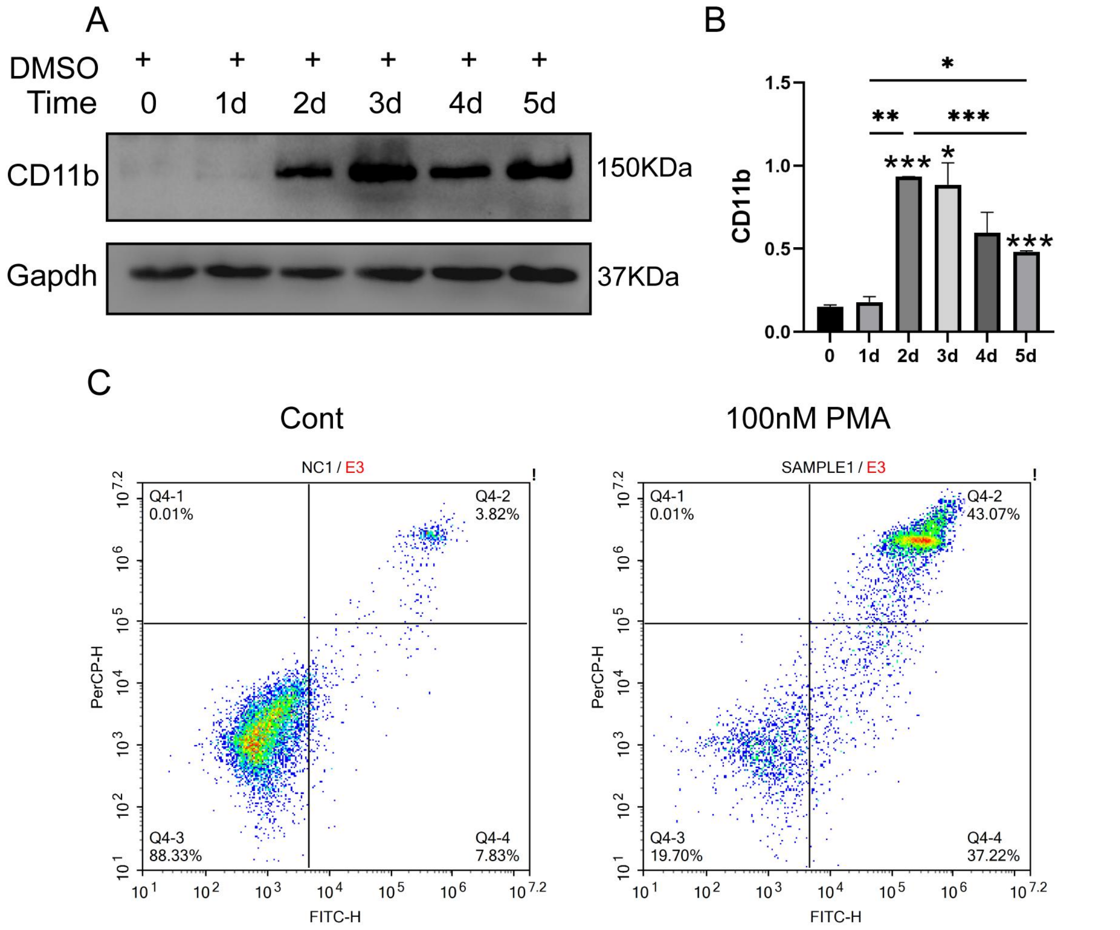

Supplementary figure 1. Differentiation of HL-60 cells into neutrophils and the induction of neutrophils apoptosis. (A, B)The expression and quantification of CD11b in HL-60 cells was verified undertreatment of 1.3% DMSO in different time point by Western blotting. (C) Measurement of apoptosis in dHL-60 cells under the treatment with 100nM PMA by flow cytometry. The percent of apoptotic cells were the ratio of cell in Q4-2 and Q4-4. *P＜0.05, **P＜0.01, ***P＜0.001.
